# HuD Regulates mRNA-circRNA-miRNA Networks in the Mouse Striatum Linked to Neuronal Development and Drug Addiction

**DOI:** 10.3390/biology10090939

**Published:** 2021-09-20

**Authors:** Michela Dell’Orco, Amir Elyaderani, Annika Vannan, Shobana Sekar, Gregory Powell, Winnie S. Liang, Janet L. Neisewander, Nora I. Perrone-Bizzozero

**Affiliations:** 1 Department of Neurosciences, University of New Mexico Health Science Center, University of New Mexico, Albuquerque, NM 87131, USA; micheladellorco@salud.unm.edu; 2 Neurogenomics Division, Translational Genomics Research Institute, 445 N. Fifth Street, Phoenix, AZ 85004, USA; aelyaderani91@gmail.com (A.E.); shobus88@gmail.com (S.S.); wliang@tgen.org (W.S.L.); 3 School of Life Sciences, Arizona State University, Tempe, AZ 85287, USA; avannan@asu.edu (A.V.); Gregory.Powell.1@asu.edu (G.P.); JANET.NEISEWANDER@asu.edu (J.L.N.)

**Keywords:** RNA-binding proteins, miRNAs, circRNAs, ceRNAs, HuD, ELAVL4

## Abstract

**Simple Summary:**

Gene expression controls all aspects of life, including that of humans. Genes are expressed by copying the information stored in the DNA into RNA molecules, and this process is regulated in part by multiple RNA-binding proteins (RBPs). One such protein, HuD, plays a critical role in the development of neurons and has been implicated in childhood brain tumors, neurodegenerative disorders (Parkinson’s, Alzheimer’s, and ALS), and drug abuse. In addition, HuD participates in neuronal remodeling mechanisms in the mature brain and promotes regeneration of peripheral nerves. HuD primarily binds to transcribed messenger RNAs, which are then stabilized for translation into proteins. However, recent studies demonstrate that HuD also regulates the expression of non-coding RNAs, such as circular RNAs (circRNAs) and microRNAs (miRNAs). In this study, we examined the role of HuD in the control of non-coding RNA expression in the mouse striatum, a brain region associated both with normal behaviors and pathological conditions such as drug abuse. Our results show that HuD regulates mRNA-circRNA-miRNA networks involved in the expression of genes associated with brain development and remodeling of neuronal connections. These findings suggest the possibility of new mechanisms controlling brain development, neurodegenerative diseases, and substance use disorders.

**Abstract:**

The RNA-binding protein HuD (a.k.a., ELAVL4) is involved in neuronal development and synaptic plasticity mechanisms, including addiction-related processes such as cocaine conditioned-place preference (CPP) and food reward. The most studied function of this protein is mRNA stabilization; however, we have recently shown that HuD also regulates the levels of circular RNAs (circRNAs) in neurons. To examine the role of HuD in the control of coding and non-coding RNA networks associated with substance use, we identified sets of differentially expressed mRNAs, circRNAs and miRNAs in the striatum of HuD knockout (KO) mice. Our findings indicate that significantly downregulated mRNAs are enriched in biological pathways related to cell morphology and behavior. Furthermore, deletion of HuD altered the levels of 15 miRNAs associated with drug seeking. Using these sets of data, we predicted that a large number of upregulated miRNAs form competing endogenous RNA (ceRNA) networks with circRNAs and mRNAs associated with the neuronal development and synaptic plasticity proteins LSAMP and MARK3. Additionally, several downregulated miRNAs form ceRNA networks with mRNAs and circRNAs from MEF2D, PIK3R3, PTRPM and other neuronal proteins. Together, our results indicate that HuD regulates ceRNA networks controlling the levels of mRNAs associated with neuronal differentiation and synaptic physiology.

## 1. Introduction

RNA-binding proteins (RBPs) play multiple roles in the post-transcriptional regulation of gene expression, from mRNA localization and metabolism to translation. These proteins are particularly important in neurons where mRNAs are transported to dendrites and axons for localized translation [1,2,3]. The neuronal RBP HuD is a member of the Hu protein family, which are mammalian homologs of Drosophila ELAV, a protein that is essential for the establishment of the neuron cell lineage [4], and one of the three mammalian ELAV-like proteins expressed in neurons (nELAVs) [5]. HuD is developmentally regulated and known to accelerate axonal outgrowth, neuronal differentiation, and nerve regeneration by regulating a large number of growth-associated genes [6,7,8]. HuD and other Hu proteins are the best-known mRNA stabilizers [9,10]. These proteins bind to AU-rich elements (AREs) in the 3′ UTR of mRNAs, thereby increasing their half-life. Binding to the same element also controls mRNA transport and local translation [11]. Furthermore, Hu proteins are known to compete with microRNAs for the regulation of gene expression [12].

Besides its role in neuronal development, HuD regulates synaptic plasticity mechanisms in mature neurons. This RBP is upregulated in the hippocampus during normal learning and memory [13,14,15], and its levels increase markedly after epileptic seizures [16,17]. Additionally, HuD has been implicated in neurodegenerative disorders (Parkinson’s, Alzheimer’s, and ALS) and substance use disorders [18,19,20,21,22,23]. HuD expression is increased by acute exposure to drugs of abuse such as cocaine [17], or during the establishment of cocaine conditioned place preference (CPP) [23]. Furthermore, mice overexpressing HuD in the nucleus accumbens also show increased expression of addiction related genes (ARG) along with increased levels of cocaine CPP and reinstatement of food-seeking behavior [23,24].

We have recently shown that HuD not only binds to and regulates mRNAs and miRNAs, but also interacts with circRNAs, a novel class of non-coding RNAs generated by back-splicing of pre-mRNAs. Interestingly, many of the circRNAs regulated by HuD are involved in synaptic plasticity and cocaine-seeking behavior, including *circHomer1* [25,26]. These HuD-regulated circRNAs were also found to form competing endogenous RNA (ceRNA) networks associated with neuronal differentiation and synaptic plasticity [26]. Increasing evidence indicates that ceRNAs can crosstalk by competing for common miRNAs, with other RNA molecules containing microRNA response elements (MREs), which are present in circRNAs, mRNAs, and other non-coding RNAs [27,28,29,30].

To better understand how HuD regulates the expression of coding and non-coding RNAs in brain regions associated with substance use disorders, we first searched for differentially expressed mRNAs, circRNAs, and miRNAs in the striatum of HuD KO mice, as this region is associated with addiction to multiple drugs of abuse [31]. We found decreased levels of mRNAs associated with addiction-related genes and behavior. Furthermore, deletion of HuD in striatal neurons altered multiple circRNAs and mRNAs interacting with the same miRNAs. Among these, a large number of dysregulated miRNAs were found to form ceRNA networks associated with mRNAs and circRNAs from genes involved in neuronal differentiation, cellular signaling, neurotransmission, and synaptic plasticity.

## 2. Materials and Methods

### 2.1. Animal Studies

HuD KO (*Elavl4*^−/−^) mice were a gift from Prof. Hideyuki Okano [32]. All experimental procedures were performed in accordance with the National Institutes of Health Guide for Care and Use of Laboratory Animals, and were approved by the University of New Mexico Health Sciences Center Institutional Animal Care and Use Committee (IACUC), protocol number 19-200933-HSC.

### 2.2. RNA Extraction

Total RNA was extracted from the striatum of adult HuD KO mice and wild type control littermates (*n* = 3 from each genotype) using TRIzol (Invitrogen, ThermoFisher, Waltham, MA. USA) and quantified using Qubit (Bio-Rad Laboratories, Hercules, CA, USA). RNA quality was determined using agarose gels, Bioanalyzer (Agilent technologies, Santa Clara, CA, USA) profiles and NanoDrop 1000 (ThermoFisher) absorbances. Aliquots of 2 µg RNA from 3 mice of each genotype were sent to Arraystar, Inc. for analysis of mRNA, circRNA, and miRNA levels as described below.

### 2.3. mRNA Arrays and qPCR Validation

The sample preparation and microarray hybridization were performed based on the Arraystar’s standard protocols (Arraystar Inc, Rockville, MD, USA). Briefly, mRNA was purified from total RNA after removal of rRNA (mRNA-ONLY™ Eukaryotic mRNA Isolation Kit, Epicentre Technologies Corporation, Madison WI, USA). Then, each sample was amplified and transcribed into fluorescent cRNA (Flash RNA Labeling Kit, Arraystar). The labeled cRNAs were hybridized onto Agilent Mouse LncRNA Array v 3.0. 8 × 60 K. Raw signal intensities were normalized using the quantile method by GeneSpring GX v12.1 (Agilent Technologies, Santa Clara, CA, USA), and low intensity mRNAs were filtered. mRNAs whose probes were Present or Marginal in at least 3 out of 6 samples were chosen for further analysis.

For mRNA qRT-PCR validation, aliquots containing 1 μg of total RNA from the striatum HuD KO and WT mice (*n* = 4) were reverse transcribed using the SuperScript II RT (Life Technologies, Carlsbad, CA, USA) following the manufacturer’s protocol. PCR amplifications were carried out with the CFX96 Touch Real-Time PCR Detection System using SYBR Green (Life Technologies) mix. Primer sequences for mouse LSAMP mRNA were obtained from PrimerBank [33]. FW Primer CAGTTGCCGCTGGTCCTAC, RV CTGTCTCACGGTGATGTTGTC (PrimerBank ID 30425330a1). GAPDH gene (FW TGTGATGGGTGTGAACCACGAGAA, RV GAGCCCTTCCACAATGCCAAAGTT) was used as the housekeeping gene to normalize values. Relative expression was determined using the comparative 2−ΔCt method [34].

### 2.4. circRNA Arrays

The sample preparation and microarray hybridization were performed by Arraystar, Inc. Briefly, total RNAs were digested with RNase R (Epicentre) to remove linear RNAs. Then, circRNAs were amplified and transcribed into fluorescent cRNA utilizing a random priming method (Super RNA Labeling Kit; Arraystar) and labeled cRNAs hybridized onto the Arraystar Mouse circRNA Array V2 (8 × 15 K, Arraystar). Raw signal intensities in the arrays were normalized using the quantile method in the R software limma package. circRNAs whose probes were Present or Marginal in at least 3 out of 6 samples were chosen for further analysis.

### 2.5. miRNA Sequencing

Total RNA was pretreated using NEBNext^®^ Poly(A) mRNA Magnetic Isolation Module (New England Biolabs, NEB, Ipswich, MA, USA) and RiboZero Magnetic Gold Kit (Human/Mouse/Rat) (Epicentre) before the RNA was used to prepare miRNA sequencing libraries with NEB Multiplex Small RNA Library Prep Set for Illumina. The DNA fragments in well mixed libraries were denatured with 0.1 M NaOH to generate single-stranded DNA molecules. These were then captured on Illumina flow cells, amplified in situ, and sequenced for 51 cycles on an Illumina NextSeq 500 platform (Illumina, Inc. San Diego, CA, USA). miRDeep2 software was used to quantify known miRNAs and predict novel miRNAs. The CPM values for the miRNAs were calculated and differentially expressed miRNAs were filtered using R package edgeR [35]. TargetScan 7.2 was used for analyses of target prediction.

### 2.6. Ingenuity Pathway Analysis

Molecular functions and cellular pathways of genes hosting mRNAs and miRNAs regulated by HuD were identified using Ingenuity Pathway software (IPA, Winter 2019 Release, Qiagen, Hilden, Germany) [36].

### 2.7. circRNA-miRNA-mRNA Interaction Analysis

**Figure 1:**
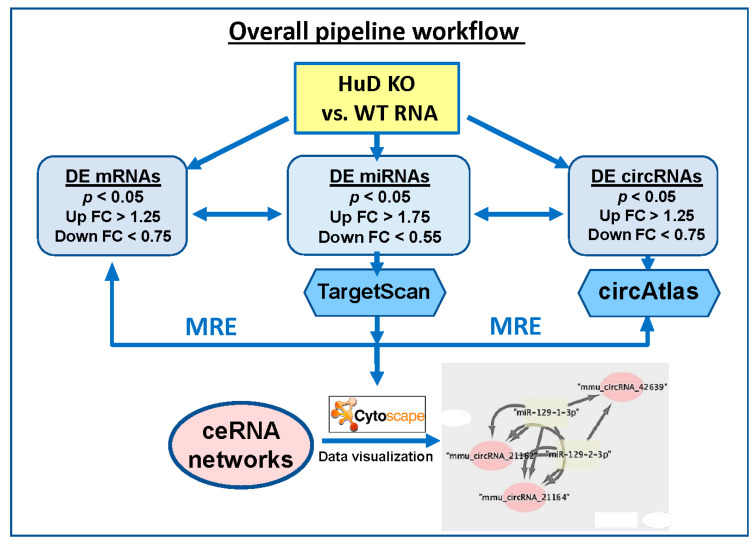
Diagram showing the flowchart of the analysis pipeline used to identify ceRNA networks of differentially expressed (DE) mRNAs, circRNAs, and miRNAs in HuD KO mouse striatum (STR). The list of mRNAs significantly downregulated in HuD KO mice was compared with those of upregulated miRNAs and downregulated circRNAs. A second analysis compared the set of upregulated mRNAs with those of downregulated miRNAs and upregulated circRNAs. See Methods and Figure S1 for more details on the analyses and network visualization using Cytoscape. MRE: miRNA recognition element.

Differentially expressed (DE) miRNAs were input into TargetScan release 7.2 (August 2018) [37], which was downloaded from GitHub [38] to identify target mRNAs that are likely regulated by these up- or down-regulated miRNAs. The parameters used for this analysis included: (a) a corresponding mRNA detection branch length 8mer ≥ 0.6, 7mer-m8 ≥ 1.8, and 7mer-1A ≥ 2.5; (b) utilization of preferentially conserved targeting [37,39]; and (c) interrogation of both canonical and non-canonical pairing of mRNA and miRNAs interaction sites, a feature that is not available on the online version of TargetScan. Unlike canonical miRNA-binding sites, which are 5′ biased on the seed sequence, non-canonical sites may be 3′ compensatory or centered; nonetheless they are known to play important roles in mRNA targeting [37,39]. Predicted mRNAs were filtered against the gene expression results from HuD KO experiments (*p* < 0.05, fold change (FC) ≥ 1.25). This analysis yielded a list of up-regulated miRNAs associated with downregulated mRNAs and a list of down-regulated miRNAs associated with up-regulated mRNAs. Separately, to identify potential circRNA targets of the same miRNAs, the lists of up- and down-regulated miRNAs was formatted into FASTA format for processing in circAtlas 2.0 [40]. Parameters for this analysis include a max binding free energy of −20 kcal/mol (using MiRanda [41]) and a circRNA–miRNA interaction score ≥ 150. Predicted circRNAs were filtered against differentially expressed circRNAs (*p* < 0.05, FC ≥ 1.25) identified using Arraystar arrays. Results from TargetScan and circAtlas analyses were integrated to identify all possible circRNA–miRNA–mRNA interactions, particularly those including: (a) upregulated miRNAs, downregulated mRNAs, and downregulated circRNAs, and (b) downregulated miRNAs, upregulated mRNAs, and upregulated circRNAs. These two combinations were selected since miRNAs are known to decrease the expression of their target mRNAs, and the levels of mRNAs and circRNAs derived from the same genes were mostly correlated in our samples (see below). Finally, these interactions were visualized in network plots generated using Cytoscape v3.8.0 (Institute for Systems Biology, Seattle, WA, USA) [42]. The overall pipeline to predict ceRNA networks is summarized in Figure 1, and a detailed description of the flowchart is shown in Figure S1.

## 3. Results

In order to identify transcriptome-wide effects of HuD deletion in the striatum, we investigated the levels of mRNAs, circRNAs, and miRNAs from HuD KO mice using the same RNA samples to probe mRNA and circRNA arrays and perform miRNA sequencing studies. Box plots demonstrate that the quartile distribution of normalized gene expression data from mRNA and circRNA arrays was the same in all KO and WT samples (Figure S2). Volcano plots of the arrays showing decreased expression of multiple RNAs in HuD KO mouse tissues were previously presented [26].

### 3.1. Alterations in mRNA Levels in the Striatum of HuD KO Mice

Analyses of Agilent arrays revealed that 3246 transcripts from 2965 genes were downregulated (Table S1a) and 3660 transcripts from 3274 genes were upregulated (Table S1b) in the striatum of HuD KO mice (FC < 0.75 or >1.25, *p* < 0.05) compared to WT controls. Comparison of the two lists revealed that 117 were in common (Table S1c), suggesting that some mRNA isoforms of those genes are downregulated, while others are upregulated in HuD KO mice. Among the upregulated mRNAs is that for the neuronal nELAV protein HuB (Elavl2). This finding is in agreement with the decreases in HuB mRNA, which is also a target of HuD, in the brains of mice overexpressing HuD [43], suggesting that these proteins are regulated in an opposite manner in neurons.

As HuD is expressed in neurons, we then compared the list of downregulated mRNAs with a set of medium spiny neuron (MSN)-expressed transcripts [44] and found that about 79% are expressed in MSNs (Table S1d). As many of the MSN-expressed mRNAs are also expressed in other cell types in the striatum, we then used the significant enrichment cut off of FDR < 0.1 utilized in Merienne et al., 2019 [44], and found that 15% of downregulated mRNAs are significantly enriched in MSNs while only 4% and 6% are enriched in astrocytes and microglial cells, respectively (Table S1d). These enrichment values are consistent with the known neuronal expression of HuD. Interestingly, the mRNA for the astrocyte-specific protein GFAP that is downregulated in HuD KO was also shown to be enriched in dopamine D2 receptor containing MSNs [44], although the protein is not normally expressed in these cells.

Given the role of Hu proteins in mRNA stability, we then examined whether deletion of HuD directly resulted in downregulation of mRNAs in HuD KO striatum using two strategies. First, we investigated if the downregulated transcripts in the KO were upregulated in HuD overexpressor (HuD OE) [23]. We found 547 mRNAs whose levels had the opposite regulation in HuD KO vs. OE mice (Table S1e), suggesting that, in the absence of HuD, these mRNAs may become destabilized, while they are stabilized by HuD overexpression. Second, we investigated how many of the downregulated transcripts are also direct targets of HuD. As shown in Table S1f, 625 downregulated transcripts are also direct targets of HuD identified by RNA-immunoprecipitation (RIP) [43], with 118 targets bidirectionally regulated in HuD KO and HuD OE tissues (Table 1 and Table S1f). In contrast, a large number of mRNAs were both upregulated in HuD KO and HuD OE, suggesting that these mRNAs are not directly regulated by HuD (Table S1g).

HuD expression is also related to cocaine-seeking behavior [23]. Thus, the set of downregulated transcripts was also compared with 388 addiction-related genes (ARGs) associated with four drugs of abuse (cocaine, alcohol, opioids, and nicotine; Table S1h) from the Knowledgebase of Addiction-related gene (KARG) mouse dataset [45]. These analyses identified in 67 downregulated ARGs in the HuD KO mice and 28 ARGs that were also upregulated in HuD OE mice such as *Bdnf, Camk2a*, *Creb1*, and *Gria2* [23] (Table 1 and Table S1h). Altogether, these results support the HuD-mediated regulation of many differentially expressed striatal mRNAs related to drug abuse.

### 3.2. Altered Biological Pathways Associated with Downregulated mRNAs

**Figure 2:**
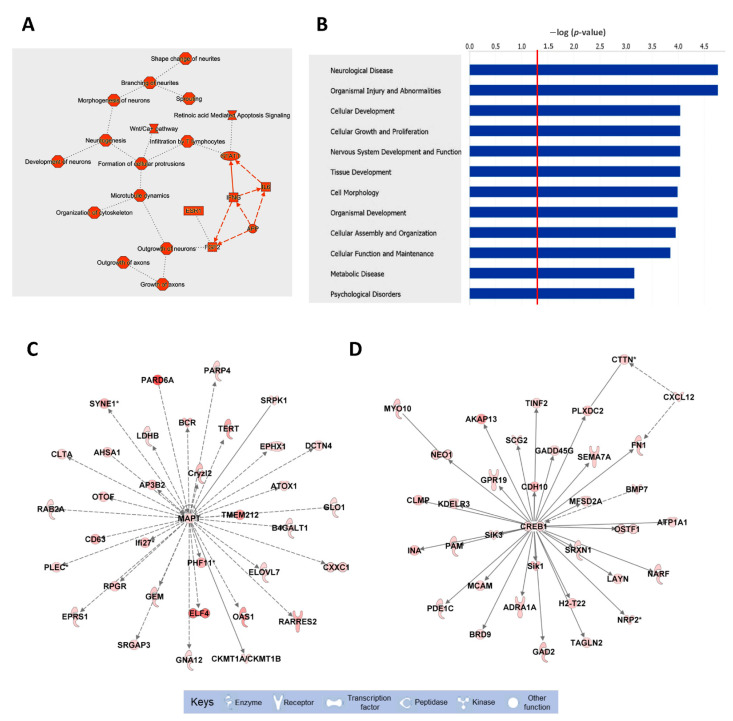
Biological pathways, diseases, and functions, and top molecular networks enriched in downregulated mRNAs in HuD KO striatum. The list of mRNAs significantly downregulated in HuD KO mice (Table S1) was imported to IPA for pathway analyses. (**A**) Top cellular pathways and (**B**) diseases and functions associated with downregulated mRNAs; the red line in (**B**) denotes a threshold of *p* = 0.05. (**C**,**D**) Top biological networks affected in HuD KO mice: (**C**) Molecules related to behavior and (**D**) cell morphology. Keys of the biological functions corresponding to the protein symbols in the networks are shown at the bottom of the figure. Note that these pathways are centered in two validated HuD targets, the MAPT and CREB1 mRNAs. The shades of red inside the protein symbols indicate the extent of downregulation of the corresponding mRNAs in HuD KO striatum, from a 5.6-fold downregulation of the PARD6A mRNA and a 4.9-fold downregulation of ELF4 mRNA, to a 1.3-fold downregulation of MAPT and CREB1 mRNAs. Solid lines denote direct interactions between proteins while indirect interactions are shown in dashed lines.

To fully understand the biological significance of these alterations, the pathways associated with the list of downregulated mRNAs were analyzed by IPA (Figure 2 and Table S2). The top functions associated with these mRNAs include neuronal morphology, neuritogenesis, and microtubule dynamics. Several biological pathways were significantly enriched in downregulated mRNAs, including neurological diseases, nervous system development and function, and psychological disorders (Figure 2B). Finally, the top two biological networks were centered on two HuD-regulated mRNAs, those for MAPT and CREB1, and contained molecules related to behavior and cell morphology (Figure 2C,D). In contrast to the downregulated mRNAs, upregulated mRNAs were associated with apoptosis of neurons, neuronal proliferation, and synaptic depression (Figure S3).

### 3.3. Dysregulation of circRNA Levels in HuD KO Striatum

Using the same RNA samples to probe circRNA arrays, we found significantly decreased levels of 4364 circRNAs from 3077 genes (FC < 0.75, *p* < 0.05) and increased levels of 4364 circRNAs from 2577 genes (FC > 1.25, *p* < 0.05) in HuD KO striatum (Table S3a and b, respectively). There were also 647 genes generating more than one circRNA, which were either upregulated or downregulated in HuD KO tissues (Table S3c). Moreover, about 60% of the downregulated circRNAs were derived from the same genes that had decreased mRNA levels, and 70% of upregulated circRNAs derived from the same genes as upregulated mRNAs (Table S3d). These results suggest that the majority of the changes in both circRNAs and mRNAs were due to changes at the level of transcription. Finally, comparison of the set of differentially expressed circRNAs with those that are bound to HuD [26] identified 64 downregulated circRNAs and 268 upregulated circRNAs that were targets of HuD [26] (Table S3e). The downregulated set included mmu_circRNA_29724, which derives from the same gene as Lsamp mRNA (see below), and mmu_circRNA_26701, deriving from the same gene as *circHomer1*, which is associated with synaptic plasticity, learning, and cocaine CPP [25,26].

### 3.4. Alterations in the Levels of miRNAs in HuD KO Striatum

**Figure 3:**
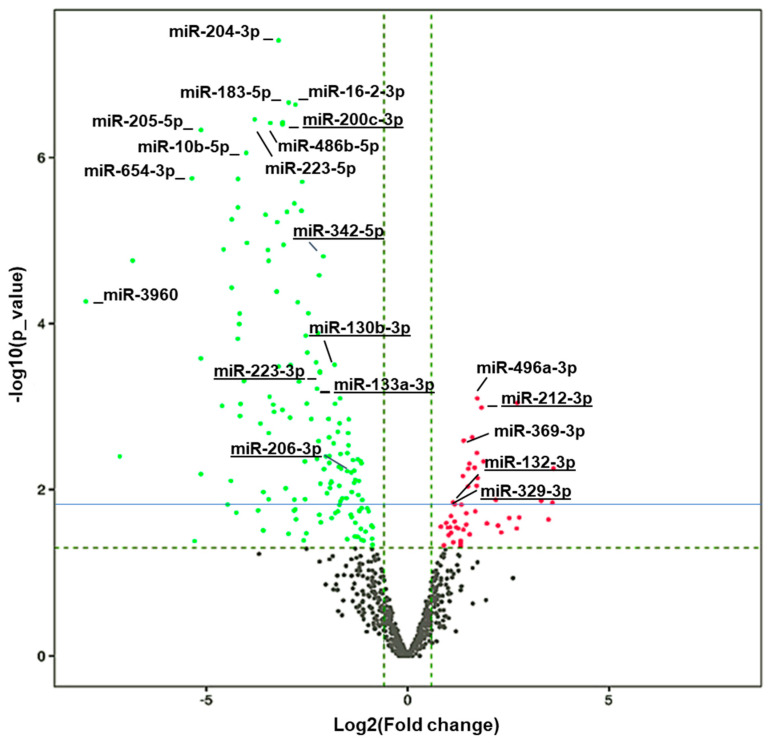
Volcano plot showing differential expressed (DE) miRNAs. Vertical dashed green lines represent the log2(FC) cut off of 2-fold, while the horizontal line green refers to the *p* value cut off of *p* = 0.05 expressed as –log(*p* value), and the solid blue line corresponds to an FDR = 0.1. Differentially downregulated miRNAs are shown in green and differential upregulated ones are shown in red. The names of some of the top upregulated and downregulated miRNAs are provided as reference. Underlined miRNAs have been previously associated with cocaine-seeking behavior [46].

The remaining amount of RNA from the samples was used for miRNA sequencing. The high quality of the data is demonstrated by a Q30 score of over 93% in all the samples (Figure S2F). A total of 161 miRNAs from 73 unique families (FC < 0.55 and *p* < 0.05) were significantly downregulated, while 148 miRNAs from 31 unique families (FC > 1.75 and *p* < 0.05) were significantly upregulated in HuD KO vs. WT mice (Table S4). A volcano plot with the names of the top altered miRNAs in HuD KO striatum is shown in Figure 3. The list of miRNAs with significant expression changes includes 15 miRNAs that were previously shown to be differentially expressed and correlated with cocaine-seeking behavior in rats exposed to environmental-enrichment housing after 21 days of forced abstinence (*miR-223-3p, miR-32-5p, miR-10a-3p, miR-200c-3p, miR-342-5p, miR-206-3p, miR-133a-3p, miR-130b-3p, miR-369-3p, miR-9b-5p, miR-326-3p, miR-325-3p, miR-329-3p, miR-212-3p,* and *miR-132-3p)* [46]. The upregulated miRNAs whose expression also significantly changed in rats displaying increased cocaine-seeking behavior include *miR-329-3p*, *miR-212-3p*, and *miR-132-3p* while the levels of downregulated miRNAs *miR-130b-3p* and *miR-133a-3p* were also altered in high vs. low cocaine-seeking rats, respectively. This list includes *miR-212-3p*, which has been previously linked to escalation of cocaine use [47].

Additionally, the levels of upregulated miRNAs *miR-369-3p, miR-9b-5p, miR-326-3p*, and *miR-325-3p,* and the downregulated miRNAs *miR-223-3p, miR-32-5p, miR-10a-3p, miR-200c-3p, miR-342-5p*, and *miR-206-3p* have been significantly correlated with levels of cocaine seeking after 21 days of forced abstinence [46].

**Figure 4:**
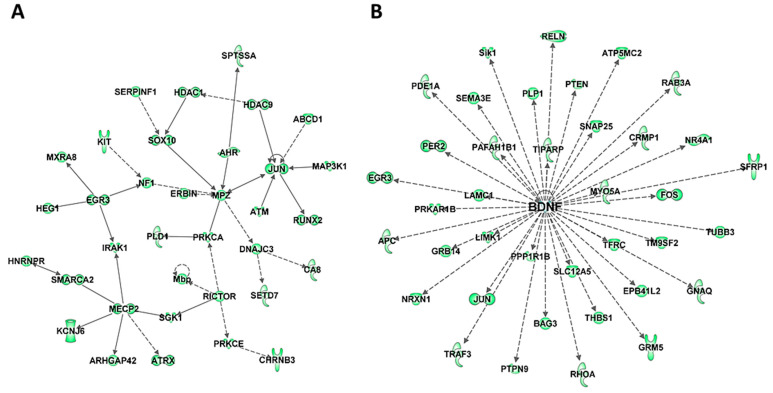
Biological networks of predicted mRNA targets of upregulated miRNAs in HuD KO striatum. The predicted target mRNAs of upregulated miRNAs (Tables S4 and S5) were analyzed by IPA. (**A**) One of the top biological networks with target mRNAs is associated with cell death and survival, neurological disease, and organismal injury and abnormalities (Table S5). (**B**) Another top biological network of predicted miRNA targets is related to BDNF. Keys of the biological functions corresponding to the protein symbols in the networks are shown at the bottom of Figure 2 and in Figure S3E.

To further understand the biological significance of DE miRNAs, their targets were examined using TargetScan context++ function of the online version of TargetScan to predict the most likely mRNA targets of these miRNAs (Table S5) and the biological pathways and networks associated with these putative targets were analyzed using IPA. Comparison of the predicted targets of upregulated miRNAs (Table S5a) with the list of significantly downregulated mRNAs in HuD KO striatum identified 364 mRNAs in common, 127 of which were also HuD targets (Table S5b). Among the 364 transcripts, there are multiple transcriptional and epigenetic regulators such as Immediate Early Genes *Jun* and *Egr3*, *Mecp2*, *Hdac* 1 and 9, and *Smarc2* (Figure 4A and Table S5c). This list also includes the ARGs and HuD targets *Bdnf* and its associated proteins (Figure 4B) and *Gria2*. In contrast, the list of predicted targets of the downregulated miRNAs (Table S5d) includes 418 upregulated mRNAs, 179 of which are predicted HuD targets including HuB mRNA (Table S5e). The two top biological networks of these targets are centered on two proteins associated with neurodegeneration, the amyloid precursor protein APP that is associated with Alzheimer’ disease and Huntingtin (HTT), a genetic mutation associated with Huntington’s disease (Figure S4 and Table S5f).

### 3.5. ceRNA Networks of miRNAs–mRNAs–circRNAs in HuD KO Striatum

**Figure 5:**
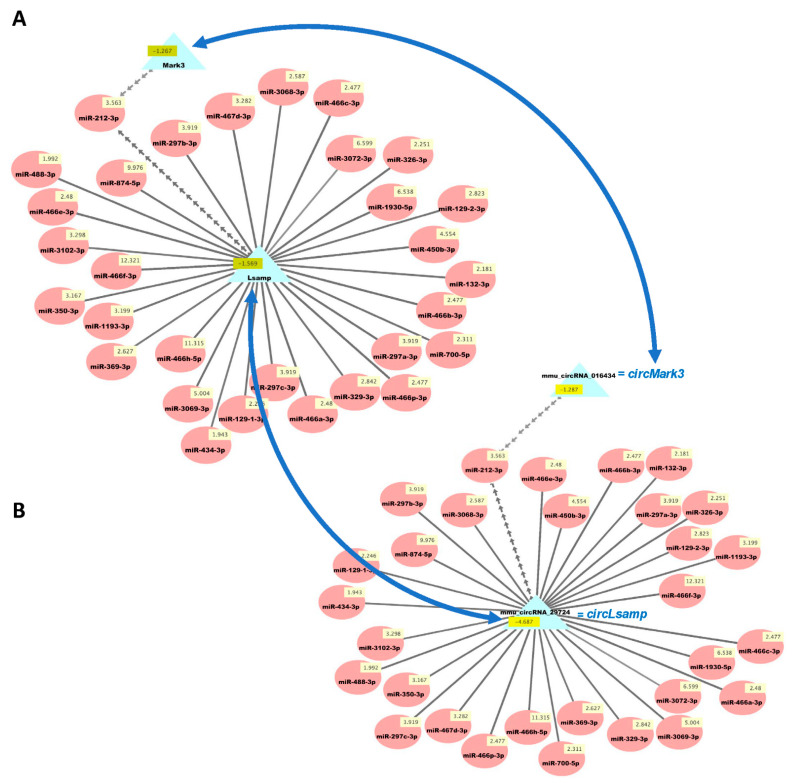
Cytoscape visualizations of predicted ceRNA interactions between significantly upregulated miRNAs in HuD KO striatum and significantly downregulated mRNAs (**A**) and circRNAs (**B**) derived from the same genes. Panels show that the mRNA and circRNA for *Lsamp* are predicted to interact with a set of 30 upregulated miRNAs, and those for *Mark3* are predicted to interact with *miR-212-3p*, as they have the same MRE sequences (Table S6). Numbers in yellow boxes represent the average levels of the miRNAs, mRNAs, and circRNAs in HuD KO mice. Multiple gray arrows indicate multiple predicted interactions and a single gray line, a single interaction. Blue arrows depict the potential competition of mRNAs and circRNAs for binding to the same miRNAs. See Methods and Figure S1 for further information.

**Figure 6:**
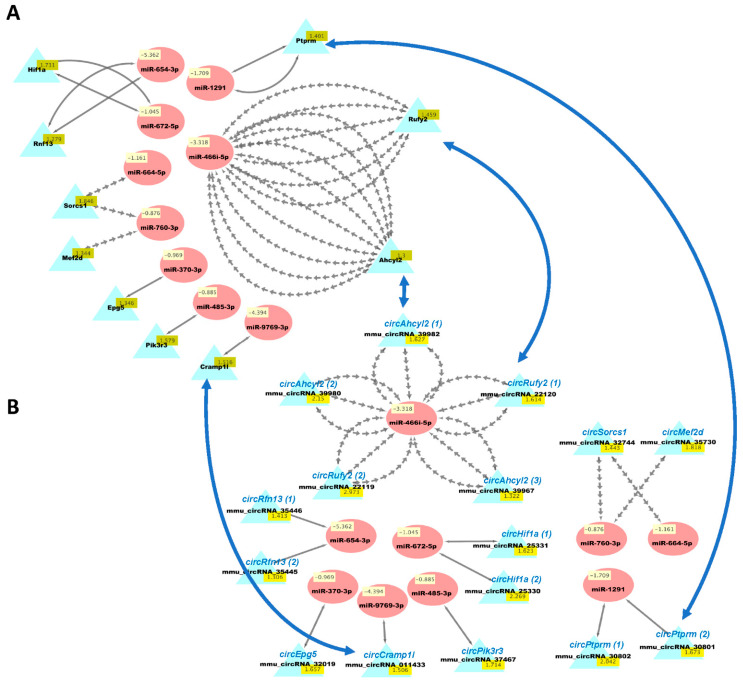
Cytoscape visualizations of predicted ceRNA interactions between significantly downregulated miRNAs in HuD KO striatum and significantly upregulated mRNAs (**A**) and circRNAs (**B**) derived from the same genes. Panels show that the mRNAs and circRNAs from same set of genes contain MRE sequences that are predicted to interact with a set of nine downregulated miRNAs (see Table S7). The names of the circRNAs are based on the genes they are derived from and are shown for comparison with the mRNAs; the numbers after the names indicate different circRNAs derived from the same gene. Multiple gray arrows indicate multiple predicted interactions and a single gray line, a single interaction. Blue arrows show examples of the potential competition of mRNAs and circRNAs for binding to and being regulated by the same miRNAs.

We then used our new analysis pipeline (Figure 1) to uncover networks of interacting ceRNAs from the same DE mRNAs, circRNAs, and miRNAs. Multiple miRNAs can bind to the same mRNA, and the same miRNAs can bind circRNAs, enabling the competition of mRNAs and circRNAs for regulation by the same set of miRNAs. As the vast majority of changes in mRNAs and circRNAs from the same genes were congruent (Table S3d), we examined the potential competition of these two types of RNAs for binding to the same miRNAs. Figure 5 shows the predicted interactions of upregulated miRNAs with both downregulated mRNAs and circRNAs in HuD KO striatum. This network included *Lsamp,* which was significantly downregulated in the Kos and is predicted to interact with 30 upregulated miRNAs (Table S6). The same miRNAs are predicted to interact with *circLsamp.* In contrast, *Mark3* mRNA and *circMark3* are predicted to interact with one miRNA, *miR-212-3p* (Figure 5). Finally, we found that nine downregulated miRNAs and a set of upregulated mRNAs and circRNAs formed other ceRNA networks involving *Hif1a, Mef2d, Pik3r3*, and other genes (Figure 6 and Table S7).

## 4. Discussion

HuD and other neuronal Hu/Elav-like (nELAVs) proteins are known to play a major role in the post-transcriptional control of gene expression in neurons. Although the vast majority of studies have focused on the direct roles of these proteins in mRNA stability [9,10], they are also known to regulate mRNA transport and translation [11]. Recent studies indicate that these proteins can also indirectly regulate mRNA levels by controlling the expression of non-coding RNAs such as circRNAs and miRNAs [26,48,49]. In this study, we demonstrated that deletion of HuD alters the levels and predicted interactions among mRNAs, circRNAs, and miRNAs in the mouse striatum, a brain region associated with locomotion, habitual learning, and substance use disorders [31].

In agreement with HuD’s role in mRNA stability, we found that a large set of downregulated mRNAs are both direct targets of HuD and upregulated in HuD OE mice, including many addiction-related transcripts [24,45]. The opposite regulation of HuD target mRNAs in KO and OE mice is consistent with the function of this RBP. However, there were multiple targets of HuD that were upregulated in the HuD KO, including the mRNA for HuB, another member of the nELAV protein family. We have previously shown that this mRNA is a target of HuD that is downregulated in HuD OE mice and upregulated in HuD KO mice [43]. One possibility for this finding is that HuB protein could be compensating for the deletion of HuD, although this was not previously observed in the same HuD/Elavl4 KO mice [32]. Another possibility is that some of the upregulated mRNAs, including HuB, could be targeted by miRNAs that are downregulated in HuD KO, as shown in Table S4, either by themselves or as part of ceRNA networks such as the one containing the HIF1α mRNA (Figure 6).

The biological pathways and functions associated with the set of downregulated mRNAs in HuD KO striatum include neuritogenesis, axonal growth, microtubule dynamics and nervous system development and function. Furthermore, the top two molecular networks from this list were centered on MAPT and the addiction-associated protein CREB1 [50], and related to neuronal morphology and behavior, which is consistent with HuD’s function in neuronal differentiation, synaptic plasticity, and cocaine seeking. Furthermore, we have previously shown that the *Creb1* mRNA is significantly upregulated in rats after a prolonged forced abstinence from cocaine-self administration, also known as the incubation period during which motivation for the drug heightens [51,52].

Among the downregulated miRNAs in HuD KO striatum, we found several miRNAs that were previously identified in the context of drug abuse, including eight miRNAs (*miR-223-3p, miR-32-5p, miR-10a-3p, miR-200c-3p, miR-342-5p, miR-206-3p, miR-133a-3p,* and *miR-130b-3p*) associated with the response to cocaine-cues after 21 days of forced abstinence [46]. In contrast, seven miRNAs associated with cocaine seeking after forced abstinence [46] (*miR-369-3p, miR-9b-5p, miR-326-3p, miR-325-3p, miR-329-3p, miR-212-3p,* and *miR-132-3p*), were significantly upregulated in HuD KO striatum. This list includes *miR-212-3p*, whose levels increase during extended access to cocaine via a mechanism involving CREB and MeCP2/BDNF signaling [47,53].

Since the discovery that mRNAs and long non-coding RNAs containing the same MREs in their sequences can compete for binding to miRNAs to form ceRNA networks [28], several studies have shown their significance in normal and pathological conditions of the nervous system [54,55,56]. These ceRNA networks add another level of complexity in the post-transcriptional control of gene expression across the entire transcriptome. Furthermore, we have recently shown that HuD plays a role in the control of both circRNA and mRNA levels, and regulates ceRNA networks during brain development and synaptic plasticity [26].

Within the set of upregulated miRNAs predicted to downregulate mRNAs and circRNAs via ceRNA interactions, we found multiple predicted interactions of 30 miRNAs with the mRNA and circRNA associated with the *Lsamp* gene. LSAMP is a plasticity-induced limbic system associated membrane protein that is required for the behavioral adaptation of mice to stressful environments [57,58]. Given that *Lsamp* is not a direct target of HuD, our findings support the notion that downregulation of this mRNA in HuD KO striatum may be caused by the direct binding of the 30 miRNAs, which are not sequestered by the decreased levels of *circLsamp.* Another predicted ceRNA interaction was associated with the *Mark3* mRNA and circRNA. Similar to the case of the *Lsamp* containing ceRNA network, the downregulation of Mark3 mRNA may be associated with the increases in the levels of *miR-212-3p*, which binds to this mRNA. However, given the complex regulatory landscape in the cells, we cannot exclude other possibilities. MARK3 is a member of the Microtubule Affinity Regulating Kinase family which is known to phosphorylate microtubule-associated proteins MAP2, MAP4, and MAPT/TAU, causing microtubule disruption in cells [59,60]. Interestingly, *Mapt* mRNA is also a target of HuD, and its levels are decreased in the striatum of HuD KO mice. MAPT is also an addiction-related protein associated with increased vulnerability to psychostimulant seeking [61] and was at the center of one of the biological networks we identified with downregulated mRNAs (Figure 2A).

Analyses of ceRNA networks associated with downregulated miRNAs identified other genes whose mRNAs and circRNAs were upregulated in HuD KO tissues. This set includes the mRNA encoding the transcription factor MEF2D, which is critical for short-term plasticity and neuronal survival [62,63]. Other transcripts in this set include those encoding the PI3K regulatory subunit gamma (PIK3R3) and protein tyrosine phosphatase PTPRM. Both PIK3R3 and PTPRM are signaling molecules that regulate a variety of cellular processes including cell growth and differentiation. PIK3/AKT signaling is critical for normal brain development [64] while PTPRM is important for cell adhesion and neurite outgrowth [65,66]. The potential role of these proteins in the synaptic changes underlying cocaine seeking remains to be investigated.

## 5. Conclusions

Overall, we found that the neuronal RNA-binding protein HuD regulates the expression of coding and non-coding RNAs in the striatum, resulting in changes in ceRNA networks associated with neuronal differentiation and synaptic remodeling. Given that HuD regulates the expression of addiction-related genes, and is associated with increased cocaine CPP, our findings suggest that the RNAs, biological pathways, and networks identified here could contribute to changes in structural and functional plasticity associated with drug-seeking behavior.

## Figures and Tables

**Table 1:** List of genes with downregulated mRNAs in HuD KO striatum that are HuD targets and ARGs.

Downregulated mRNAs that are upregulated in HuD OE mice and HuD targets ^1^ (118)	*Aebp2, Ahnak, Akap8, Akap9, Anxa2, Ap3b2, Aqp9, Arf6, Arhgef10, Arhgef10l, Asph, Auts2, Axin1, Bach1, Baz1b, Bcl9, Bdnf, Bicc1, Bicd1, Brdt, Btrc, Cab39l, Cables1, Camk2a, Cfdp1, Cnn3, Col25a1, Copa, Creb1, Csnk1g1, Cyhr1, D10Wsu102e, Ddx54, Denr, Dst, Ebna1bp2, Eif2s2, Eif4g1, Eif4g3, Elavl4, Elovl5, Elovl7, Eps15, Etfdh, Ewsr1, Fndc3a, Fus, Gak, Galnt7, Gba2, Gli3, Gpm6a, Gria2, Gsk3b, Hdac9, Hells, Hmgn1, Hook2, Igf1, Impad1, Iqgap1, Iqsec2, Irf2, Kcnn2, Kif5b, Lmnb1, Lrrc19, Luc7l2, Mapk8ip3, Mcm3, Mgrn1, Mitf, Mpdz, Mpp6, Mta3, Myo1d, Nasp, Nfia, Ophn1, Paip1, Pik3r1, Plcl1, Ppp1r2, Ppwd1, Prrx1, Rab8b, Rapgef3, Rapgef5, Rbm8a, Rbms3, Rbx1, Rdh12, Rere, Rhoc, Rps6ka3, Rrm1, Scn1a, Sdha, Skil, Slc25a30, Slc8a1, Ss18, Steap1, Tardbp, Tcf4, Tfdp2, Tmpo, Tnfaip2, Tom1l2, Top1, Tpm1, Yap1, Ybx1, Zbtb7a, Zkscan1, and Zmat2*
Downregulated mRNAs that are also ARGs ^2^ (67)	*Abat, Aldoc, Apod, Apoe, Atxn1, Bcl2l1, Bdnf, Btg3, Camk2a, Capzb, Cldn5, Cnp, Col1a2, Creb1, Cxcl12, Eno1, Enpp2, Erbb3, Fabp7, Fn1, Gadd45g, Gars, Gclc, Gfap, Gpm6b, Gria2, Homer2, Hspa1a, Hspa1b, Igfbp2, Jun, Kcnma1, Lamp1, Maob, Mapt, Mbp, Mobp, Mog, Mpdz, Nefl, Nfia, Nptxr, Nr4a3, Nsmaf, Ntrk2, Osbpl1a, Pam, Parp4, Pdia3, Pglyrp1, Ppp1r2, Ppp2r5c, Pura, Rad23b, Rpl23a, Scg2, Schip1, Sfpq, Slc3a2, Smpd2, Sod2, Tmed10, Tnfrsf9, Tra2a, Tubb2b, Ube2m, and Vdac1*
Downregulated mRNAs that are both HuD targets and ARGs ^3^ (32)	*Abat, Aldco, Apoe, Atxn1, Bdnf, Camk2a, Col1a2, Creb1, Gria2, Cxcl12, Enpp2, Fn1, Gars, Gclc, Gfap, Gria2, Homer2, Kcnma1, Lamp1, Mpdz, Nfia, Pam, Pdia3, Ppp1r2, Pura, Rpl23a, Slc3a2, Sod2, Tra2a, Tubb2b, Ube2m, and Vdac1*

^1^ The list of HuD target mRNAs that are both significantly downregulated in the striatum of HuD KO and upregulated in HuD OE mice [23]. ^2^ The list of addiction-related genes (ARGs) that are significantly downregulated in HuD KO mice and are associated with drug abuse [45]. ^3^ The same list was compared with the list of HuD target mRNAs [43]. A total of 32 genes in common were identified. The underlined genes belong to a set of behavior-associated genes [23]. The numbers in parentheses indicate the number of genes in each list.

## Data Availability

The datasets presented in this study can be found at the Gene Expression Omnibus (GEO) database, available at the National Center for Biotechnology Information (NCBI), National Institutes of Health, USA . The accession numbers for the mRNA and circRNA microarray data are GSE153406 and GSE153387, respectively, and the accession number for the miRNA-sequencing data is GSE179652.

## Supplementary Materials

The following are available online at https://www.mdpi.com/article/10.3390/biology10090939/s1, Figure S1: Detailed workflow for ceRNA analyses pipeline. Figure S2: QC analyses of RNA samples, box plots of the mRNA and circRNA array normalized data and miRNA-seq Q30 scores. Figure S3. Biological pathways, diseases, and functions, and top molecular networks associated with upregulated mRNAs in HuD KO striatum. Figure S4. Validation of the levels of Lsamp mRNA in the arrays using qRT-PCR. Figure S5: Biological networks of predicted mRNA targets of downregulated miRNAs in HuD KO striatum. Table S1: Differentially expressed mRNAs in HuD KO striatum. Table S2: Biological pathways, diseases, and function associated with downregulated mRNAs. Table S3: Differentially expressed circRNAs in HuD KO striatum. Table S4: Differentially expressed miRNAs in HuD KO striatum. Table S5: Predicted targets of upregulated and downregulated miRNAs in HuD KO. Table S6: UP_miRNA_&_DOWN_mRNA_&_DOWN_circRNA list for ceRNA network analysis. Table S7: DOWN_miRNA_&_UP_mRNA_&_UP_circRNA list for ceRNA network analysis.
