# Supplementary figures and images for: HuD Regulates mRNA-circRNA-miRNA Networks in the Mouse Striatum Linked to Neuronal Development and Drug Addiction
Source: Biology (Basel). 2021 Sep 20;10(9):939. doi: 10.3390/biology10090939 (PMC8468275; doi:10.3390/biology10090939)

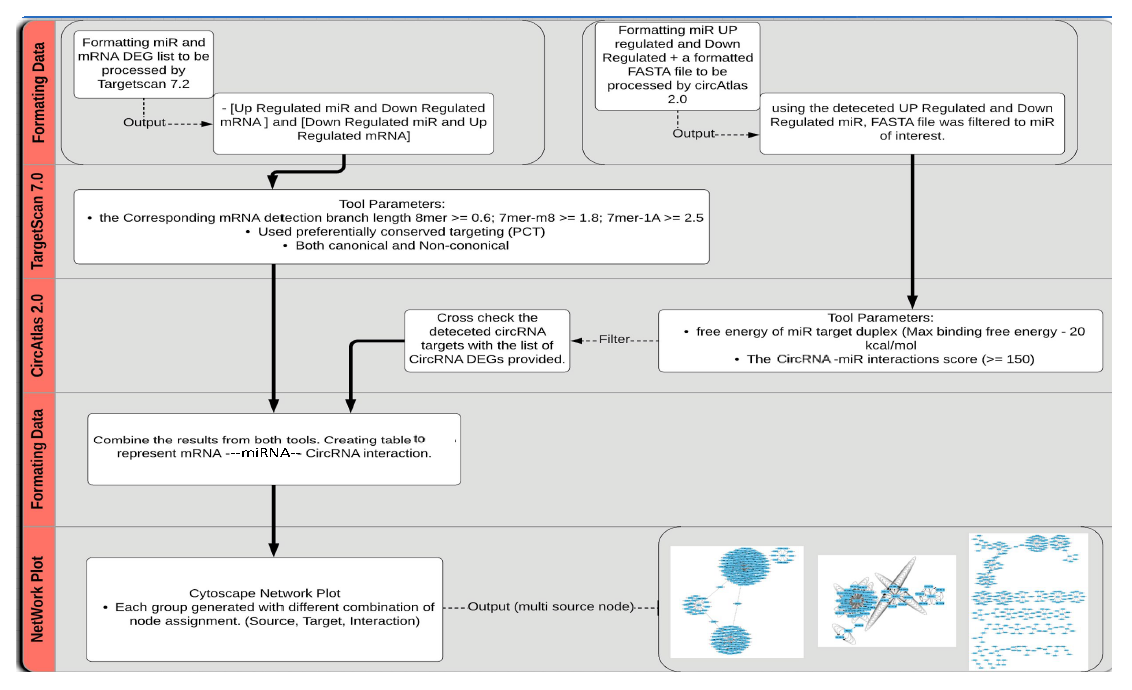

Supplement: Supplementary file 1 [file biology-10-00939-s001.zip › biology-1322900-supplementary/Second REV Supplementary Figures/Figure S1.tif]

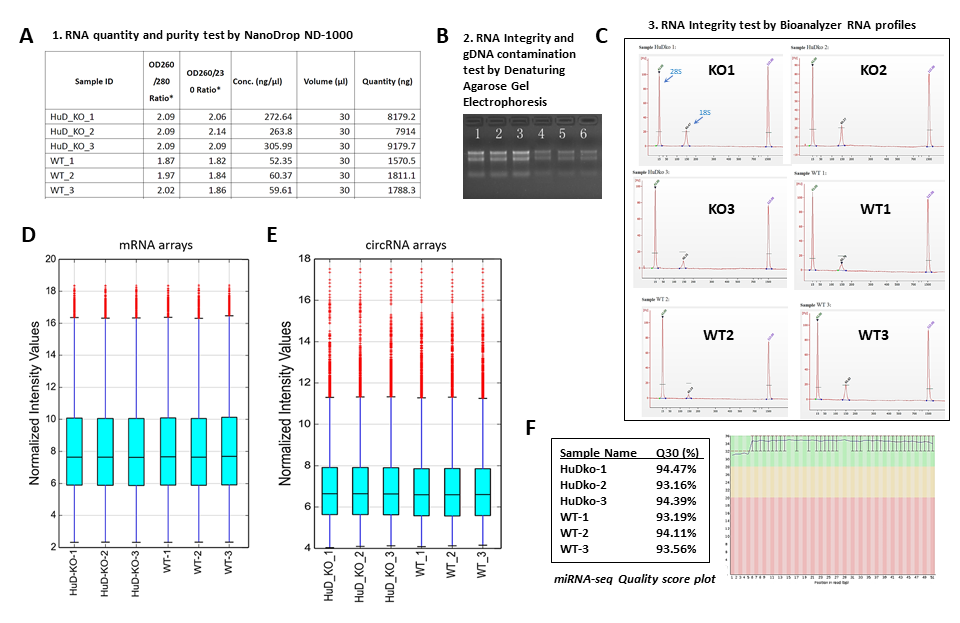

Supplement: Supplementary file 1 [file biology-10-00939-s001.zip › biology-1322900-supplementary/Second REV Supplementary Figures/Figure S2.tif]

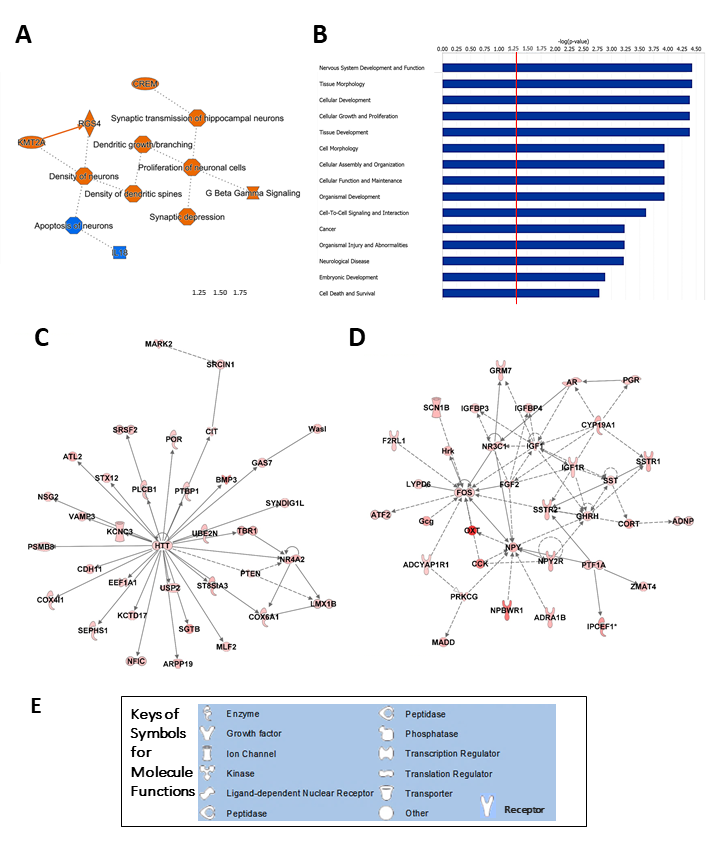

Supplement: Supplementary file 1 [file biology-10-00939-s001.zip › biology-1322900-supplementary/Second REV Supplementary Figures/Figure S3.tif]

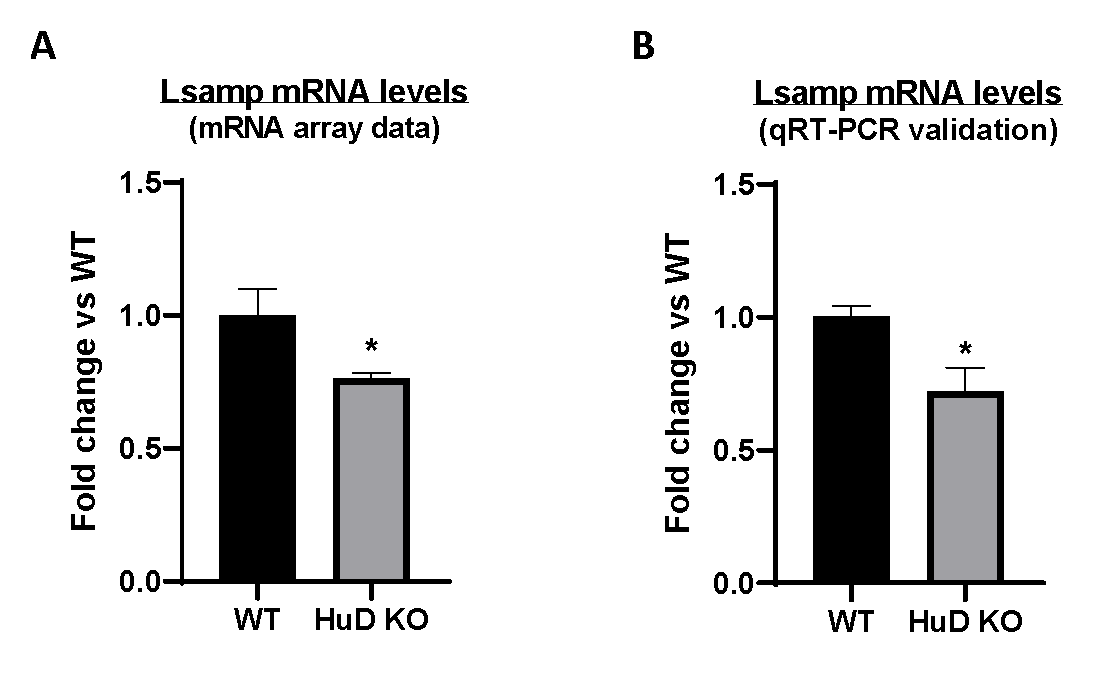

Supplement: Supplementary file 1 [file biology-10-00939-s001.zip › biology-1322900-supplementary/Second REV Supplementary Figures/Figure S4.tif]

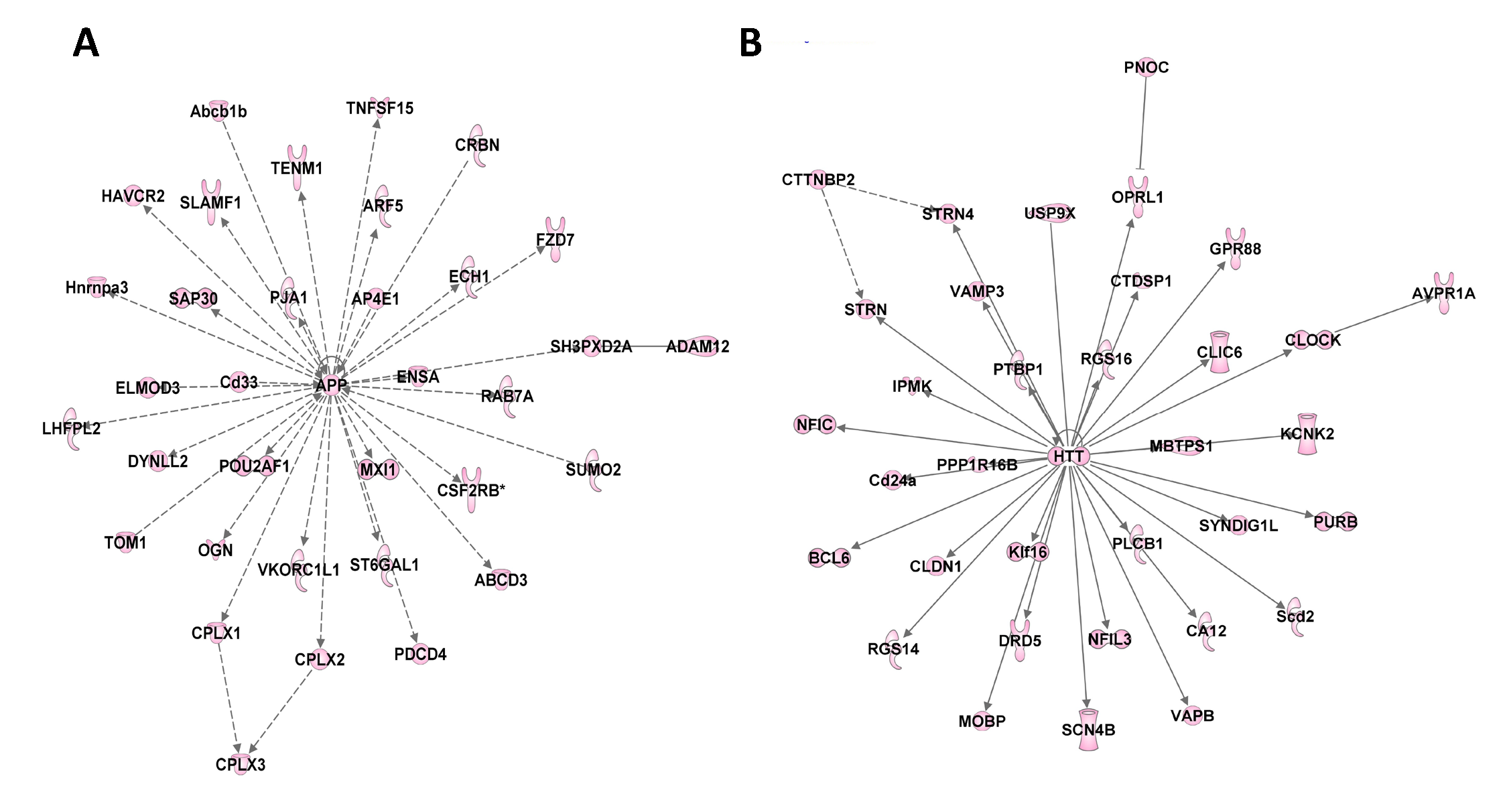

Supplement: Supplementary file 1 [file biology-10-00939-s001.zip › biology-1322900-supplementary/Second REV Supplementary Figures/Figure S5.tif]
